# Attachment-related trauma markers and symptom severity in Afghan refugees

**DOI:** 10.1192/bjo.2026.12057

**Published:** 2026-08-10

**Authors:** Thomas Egger, Anna Buchheim, Britta Dumser, Manuela Gander

**Affiliations:** Institute of Psychology, University of Innsbruck, Innsbruck, Austria; Department of Psychotherapy and Social Counselling for Adults, Refugio Munich, Munich, Germany; Department of Child and Adolescent Psychiatry, Medical University of Innsbruck, Innsbruck, Austria

**Keywords:** Attachment, trauma, Afghanistan, refugees, trauma markers

## Abstract

**Background:**

Afghan refugees experience high levels of cumulative trauma and psychosocial adversity, yet attachment-related trauma has rarely been examined at the narrative level. Narrative attachment assessments may capture culturally embedded expressions of distress beyond post-traumatic stress disorder (PTSD)-focused symptom models.

**Aims:**

This study examined narrative attachment-related trauma markers in Afghan refugees receiving out-patient psychological treatment. It investigated whether trauma marker frequency differed between resolved and unresolved attachment representations and whether it was associated with psychological symptom severity.

**Method:**

In an exploratory cross-sectional study, 40 Afghan refugees (mean age = 25.4 years; 75% male) receiving out-patient psychological treatment completed the Adult Attachment Projective Picture System, a narrative-based attachment interview. Trauma markers were identified and quantified within the narratives. Associations with attachment representations (resolved versus unresolved) and psychological symptom severity were examined in a subsample with available self-report data (*n* = 27).

**Results:**

Participants with unresolved attachment representations showed higher trauma marker frequencies than those with resolved representations. Trauma marker frequency was positively associated with depressive symptoms and overall psychological distress (Spearman’s effect size estimate (*r*) = 0.40–0.51; 95% bias-corrected and accelerated (BCa) confidence intervals excluding zero) but not with PTSD symptom severity.

**Conclusions:**

Trauma markers captured clinically relevant distress in Afghan refugees beyond PTSD-specific symptomatology, particularly depressive symptoms and general psychological burden. Narrative, attachment-based approaches may be especially valuable in clinical encounters in which distress is conveyed implicitly, relationally, or in culturally contextualised ways rather than through explicit symptom reporting. Culturally sensitive applications of such methods, supported by well-trained interpreters, are essential for clinical understanding and meaningful clinical engagement.

Afghan refugees – one of the largest refugee populations worldwide^1^ – remain markedly underrepresented in clinical attachment research, despite robust evidence that attachment is strongly associated with mental health outcomes^2^ and that high prevalence rates of depression and post-traumatic stress disorder (PTSD) have been consistently reported across refugee populations.^3^ Since the Taliban takeover in August 2021, the need for psychological support among the Afghan population has increased substantially, while mental healthcare has simultaneously been deprioritised at both individual and governmental levels.^4^ Afghan refugees living in Europe also experience a wide range of physical and psychological health burdens, including infectious and non-communicable diseases as well as high levels of psychological distress.^5^

Given evidence linking attachment insecurity with post-migration stress and post-traumatic stress symptoms in refugees,^6^ a more differentiated understanding of attachment may help elucidate mental health vulnerabilities in this population, including Afghan refugees. To date, however, only our previous study has examined attachment in Afghan refugees undergoing psychological treatment using a validated attachment interview.^7^ Whereas our earlier work focused on categorical attachment classifications and their associations with psychological symptom severity, the present study shifts the focus towards the narrative expression and severity of attachment-related trauma, taking into account cultural and communicative factors that may shape how attachment-related distress is articulated. Narrative approaches to adult attachment assessment, such as the Adult Attachment Projective Picture (AAP), rely on the verbal communication of attachment-related content.^8^ Accordingly, it remains unclear how attachment-related and potentially traumatic content is communicated by Afghan refugees. The present study aims to address this gap.

## Adult attachment and clinical correlations of psychopathology

Attachment – as conceptualised by John Bowlby – is a biologically based system between a child and their primary caregiver, with attachment behaviours aimed at restoring proximity and safety in situations of separation.^9^ Over the course of development, individuals form internal working models of their own attachment and that of others in the form of attachment representations, which guide the anticipation, interpretation and regulation of interpersonal relationships.^10^ In adulthood, attachment representations are commonly conceptualised using either categorical or dimensional approaches. Dimensional attachment is most often measured using self-report questionnaires that assess consciously accessible relationship attitudes and experiences.^11^ Categorical instruments such as the Adult Attachment Interview assess attachment using an interview-based format^12^ and are considered better suited to accessing unconscious attachment-related processes, particularly in clinical contexts.^11^

Adult attachment classifications are derived from the coherence and organisation of the interviewee’s narrative in response to attachment-related prompts. Four attachment classifications are commonly distinguished: secure (F), dismissing (Ds), preoccupied (E) and unresolved (U).^8^ In the adult attachment literature, unresolved classifications are also reported using abbreviations such as ‘U/d’, and the terms ‘unresolved’ and ‘disorganised’ are often used interchangeably.^13^ In some studies, unresolved classifications are reported together with ‘cannot classify’ (CC).^14^ For simplicity, the present paper refers to these classifications under the umbrella term ‘unresolved’, unless finer distinctions are relevant. Within this framework, the concept of defensive exclusion has proven particularly useful for capturing individual differences in mental attachment representations and for assigning attachment status.^8^

Unresolved attachment differs fundamentally from the other attachment categories. It is characterised by an inability to integrate and reorganise narratives containing markers of segregated systems. A segregated system refers to an intensified defensive process through which distressing memories and affects associated with a threatening attachment relationship are split off from conscious awareness. Individuals with an unresolved attachment representation may become temporarily overwhelmed by attachment-related fears and threat and are unable to regain narrative coherence, even though narrative content and defensive processes may resemble those observed in other attachment groups.^15^ This qualitative difference allows the remaining three attachment classifications to be grouped as ‘resolved’.^8^

In adulthood, unresolved attachment appears particularly prominent in clinical samples and is considered to be related to traumatic experiences.^16^ Emerging evidence suggests that these associations also apply to refugee populations,^17^ with high rates of unresolved attachment reported among refugees,^18^ including Afghan samples.^7^

## Cultural influences on the communication of attachment and trauma

Despite its biological and universal foundations, attachment patterns are shaped not only by caregiving experiences, but also by cultural contexts that organise parenting roles, caregiving arrangements and social support.^19^ Moreover, a substantial proportion of attachment research is based on ‘WEIRD samples’ (Western, Educated, Industrialised, Rich and Democratic).^20^ In refugee research in particular, migration-specific adaptations of attachment instruments have yielded preliminary but promising results.^21^

Within the context of psychological distress and attachment, communication style plays a critical role. Many Afghan refugees may originate from collectivist cultural contexts^22^ in which emotional expression is more strongly regulated to maintain social harmony.^23^ Refugees often report that psychological distress is addressed only once clinicians actively establish a comfortable atmosphere and directly inquire about trauma-related experiences.^24^ This underscores the need for context-sensitive interventions informed by Afghan perspectives on mental health and psychosocial well-being.^25^ At a linguistic level, Afghan individuals often use idiomatic expressions to describe psychological distress, and there is no direct equivalent term for PTSD.^26^

Research on trauma and PTSD in Afghan populations presents an ambivalent picture: while PTSD appears to demonstrate construct validity, war-related trauma exposure is not more strongly associated with depression, anxiety and general psychological distress than with PTSD specifically.^27^ Trauma among Afghan refugees should therefore be conceptualised in a holistic manner that is embedded within the individual life narrative, with attachment playing a central role.

Empirical evidence indicates that higher post-traumatic burden is associated with a more frequent occurrence of trauma-related content in narratives, particularly references to death and dying.^28^ Such content is recalled with greater emotional intensity, occurs more frequently both voluntarily and involuntarily, and assumes a central position within the autobiographical life narrative, especially in individuals with PTSD.^29^ The active narrative processing of traumatic life experiences is therefore a central therapeutic mechanism, as reflected in Narrative Exposure Therapy,^30^ which has demonstrated effectiveness in refugee populations.^31^ From an attachment perspective, narrative processing is also relevant because attachment representations are reflected in the organisation, coherence and regulation of attachment-related narratives. Thus, both trauma-focused narrative approaches and narrative attachment assessments emphasise how distressing experiences are organised or remain fragmented within personal narratives.

Against this background, the present study investigated attachment and trauma using a construct-based coding dimension derived from the AAP coding system.^8^ In this study, narrative attachment-related trauma markers refer to markers of segregated systems in AAP narratives, a concept rooted in Bowlby’s theory of defensive exclusion.^32^ They are narrative indicators of attachment-related threat, fear, helplessness, abandonment, failed protection, isolation or constriction.^8^ Trauma markers may occur across attachment classifications and do not represent externally verified traumatic events, but rather narrative expressions of attachment-related traumatic content within the AAP. Accordingly, the study addressed the following research question: how frequently do trauma markers occur in the AAP narratives of Afghan refugees, and how is trauma markers frequency associated with attachment representations and psychological symptom severity?

To our knowledge, this study is among the first to systematically examine attachment-related trauma markers in Afghan refugees and to investigate their associations with attachment representations and psychological symptom severity using a narrative-based attachment interview.

Two exploratory hypotheses were formulated. First, participants with unresolved attachment representations were expected to show higher trauma marker frequency than participants with resolved attachment representations. Second, higher trauma marker frequency was expected to be associated with greater psychological symptom severity, particularly depressive symptoms and general psychological distress, and exploratively with PTSD symptom severity.

## Method

### Study design

This exploratory cross-sectional study investigated the narrative expression and severity of attachment-related trauma in a clinical out-patient sample of Afghan refugees undergoing psychological treatment. Building on a previous study that focused on categorical attachment classifications and their associations with psychological symptom severity, the present study adopted a narrative-analytic approach, examining the frequency of trauma markers and their associations with attachment representations and psychological distress.

### Participants and recruitment

Participants were Afghan refugees with diverse regional and ethnic backgrounds receiving out-patient psychological treatment at Refugio Munich, a specialised psychosocial treatment centre for refugees in Munich. Ethnicity was self-identified by participants as Afghan; no further sub-ethnic categorisation was imposed to avoid over-stratification of a small sample. Recruitment took place between September 2022 and July 2023. All participants were referred to the study by their treating clinicians and were approached consecutively.

The total sample comprised 40 Afghan refugees who completed the AAP assessment. Despite the use of culturally sensitive diagnostic procedures and an approach aligned with World Health Organization (WHO) recommendations for refugee mental health care,^33^ not all participants were able to complete questionnaire-based assessments at treatment entry. For analyses involving psychological symptom severity, only participants with available questionnaire data were included, resulting in a subsample of 27 participants. This subsample was nearly balanced with regard to attachment status (resolved: *n* = 14; unresolved: *n* = 13). Descriptive comparisons indicated no meaningful differences in age or gender between participants with complete and incomplete questionnaire data. However, given the small sample size, no formal dropout analysis was conducted. Reduced data availability primarily reflected the clinical non-feasibility of questionnaire completion due to high levels of psychological distress and may nevertheless have introduced selection bias into the symptom analyses. This challenge is well documented in refugee mental health research and occurs in the context of commonly reported barriers to questionnaire-based assessment, including stigma, language and communication difficulties, cultural and religious factors, and structural access constraints.^34^ There were no additional exclusion criteria beyond the inability to complete the assessment procedures.

### Procedure

The study received ethical approval (Nr. 05/2022) from the Board for Ethical Questions of the University of Innsbruck. Data collection was conducted within an out-patient treatment setting at the treatment centre. Participants received a compensation of €15.

After providing written informed consent, the attachment interview (AAP) was conducted as part of a structured research procedure while participants were receiving ongoing psychological treatment. The attachment interviews were conducted by the first author, a male European clinical psychologist working at the treatment centre and trained in culturally sensitive and trauma-informed approaches with refugee populations. Assessments were conducted in the participants’ preferred language (Dari or Pashto), with the assistance of trained interpreters when required. To mitigate potential gender- and culture-related influences on narrative expression, interpreters were carefully selected with regard to gender and cultural background, and matched to participants whenever possible.

Baseline psychological symptom severity had been assessed at treatment entry as part of routine clinical diagnostics at the treatment centre, using self-report questionnaires such as the Hopkins Symptom Checklist–25 (HSCL-25) and Post-traumatic Stress Disorder Checklist for DSM-5 (PCL-5). With participants’ permission, these questionnaire-based baseline symptom data were extracted from clinical records and linked to the AAP assessment. The AAP assessment was not part of routine clinical diagnostics, but was conducted separately at a later point during ongoing psychological treatment. The primary narrative variable of the present study, trauma marker frequency, was derived from AAP narratives and not from clinical records. Questionnaire measures were therefore not administered concurrently with the AAP assessment.

All assessments took place in a quiet and private setting within the treatment centre. Participants were informed that participation was voluntary and that their decision would not affect their ongoing treatment. Given the emotionally evocative nature of narrative attachment interviews, particular attention was paid to participants’ emotional well-being. In case of elevated emotional distress following the interview, additional therapeutic support was available within the ongoing treatment context.

### Measures

#### AAP

Adult attachment representations were assessed using the AAP, a semi-structured, narrative-based assessment designed to activate the attachment system and elicit narratives reflecting internal working models of attachment. The AAP consists of a set of attachment-related picture stimuli depicting scenes involving separation, solitude, illness, threat and loss. Participants are asked to describe what is happening in each scene, what the depicted characters might be thinking or feeling and how the situation might unfold. Responses are intended to capture implicit attachment-related processes as they are expressed in narrative form.^8,15^

Narratives were audio-recorded and transcribed verbatim for subsequent coding and analysis. Attachment representations were classified according to established coding guidelines into secure, dismissing, preoccupied or unresolved categories.^8^ In line with the aims of the present study, attachment classifications were subsequently grouped into resolved (secure, dismissing, preoccupied) and unresolved categories.

The AAP has demonstrated good reliability and strong convergent validity with the Adult Attachment Interview (AAI)^35^ and has been applied in refugee-related clinical contexts.^36^

#### Coding of attachment-related trauma markers

To assess attachment-related traumatisation beyond categorical attachment classifications, a construct-based coding approach was applied to quantify trauma-related content in AAP narratives. Specifically, the analysis focused on one step of the AAP narrative coding procedure, namely the identification of markers of segregated systems.^8^ The concept of segregated systems derives from Bowlby’s theory of defensive exclusion.^32^ In the AAP, these markers indicate frightening, threatening or traumatic attachment-related material that may enter the narrative when defensive segregation begins to fail.^37^

Markers of segregated systems include themes of fear, helplessness, failed protection, abandonment, dangerous events, severe isolation, dissociative or eerie imagery and intrusions of frightening or traumatic material. They may occur across all attachment classifications and are first identified before being evaluated as resolved or unresolved. Resolution refers to the capacity to integrate or contain threatening material through organised attachment representations, for example by seeking protection, relying on internal resources or engaging in protective action. If such resolution is absent, the material remains unresolved and continues to be marked by fear, helplessness, abandonment or vulnerability. Constriction constitutes a distinct form of segregated systems marker and refers to a restricted response style in which individuals avoid engaging with the picture; it may therefore indicate unresolved segregated material even in the absence of explicit traumatic themes.^8^

For the purposes of the present study, resolved and unresolved markers of segregated systems were counted collectively as trauma markers, irrespective of their resolution status. Thus, trauma marker frequency operationalised the amount of attachment-related traumatic content within the narratives and was not equivalent to an unresolved attachment classification. This dimensional approach complements categorical attachment classifications and allows for the examination of individual differences in the narrative expression of attachment-related trauma.

Building on previous AAP-based work, trauma markers included references to (a) danger or failed protection, such as abandonment or threat without protection; (b) helplessness or emotional collapse; (c) isolation or emptiness; (d) disturbing or fear-inducing content; (e) intrusive or overwhelming fear-related material; and (f) narrative constriction, defined as an inability to continue or complete the narrative due to overwhelming attachment-related distress.^38^ This included both explicit phrases, such as expressions of hopelessness or annihilation, and emotionally condensed single words indicating a breakdown of attachment-related regulation.

All attachment narratives were systematically reviewed, and trauma markers were identified and counted. The total number of trauma markers was summed to create a dimensional index of attachment-related trauma severity, with higher scores indicating greater attachment-related traumatic content. This approach is grounded in evidence that greater trauma-related narrative content is associated with more severe post-traumatic psychopathology.^28^ Traumatic memories in PTSD are likewise marked by heightened emotional intensity and autobiographical centrality.^29^

For the classification of attachment representations (secure, dismissing, preoccupied, unresolved), all AAP transcripts were coded by the primary certified AAP rater, and 50% were independently double-coded by one of two additional certified AAP raters. Cohen’s kappa for interrater reliability between the primary rater and the additional raters was *κ* = 0.80 across the double-coded transcripts, indicating good agreement. In cases of disagreement, transcripts were reviewed by a third certified AAP rater. Although no separate interrater reliability coefficient was calculated for trauma marker coding, consistency was supported by linking trauma marker identification to the AAP coding evaluation underlying the final attachment classification. For transcripts that were double-coded or reviewed by a third rater, trauma markers were therefore identified in the coding version on which the final attachment classification was based. This procedure ensured consistency between attachment classification and trauma marker coding.

This coding approach has previously been shown to capture clinically meaningful differences in attachment-related trauma severity between psychiatric and control samples.^38^

In the present study, it was adapted to translated AAP transcripts from an adult refugee sample, with careful consideration of culturally diverse narrative expressions of distress.

#### Symptom measures

Psychological symptom severity was assessed at treatment entry as part of routine clinical procedures using validated self-report questionnaires administered in an assisted format. Assessments were conducted by trained clinicians, with the support of professional interpreters when required, to ensure comprehension and accurate responding. Questionnaires were either completed in validated written Persian versions or administered orally with interpreter assistance in Dari or Pashto, following forward–backward translation procedures in accordance with established best-practice guidelines.^39^

Depressive symptoms and general psychological distress were measured with the HSCL-25,^40^ which assesses symptoms of depression and anxiety on a four-point Likert scale and has demonstrated applicability in refugee and asylum-seeking populations.^41^

Post-traumatic symptom severity was assessed using the PCL-5,^42^ a 20-item self-report measure assessing core PTSD symptoms according to DSM-5 criteria. Items are rated on a five-point Likert scale, with higher scores indicating greater symptom severity. The PCL-5 has also demonstrated applicability in refugee populations.^43^

### Statistical analysis

Statistical analyses were conducted using IBM SPSS Statistics for macOS (version 30.0; IBM Corp., Armonk, New York, USA; https://www.ibm.com/products/spss-statistics). Given the exploratory nature of the study, the small sample sizes and non-normal distributions of key variables, non-parametric statistical methods were applied throughout. Effect sizes were interpreted descriptively given the exploratory nature of the study.

To test Exploratory Hypothesis 1, differences in attachment-related trauma marker expression between participants classified as resolved and unresolved were examined using two-tailed Mann–Whitney *U* tests (*N* = 40). Trauma marker frequency was operationalised using three complementary indices: (a) the total number of trauma markers (sum), (b) trauma marker density (number of trauma markers relative to total word count) and (c) trauma marker coverage (number of attachment stimuli containing at least one trauma marker). These indices were treated as alternative operationalisations of the same underlying construct. Accordingly, the Holm–Bonferroni procedure^44^ was applied to control the familywise error rate across the three comparisons. To quantify the magnitude and precision of group differences, Hodges–Lehmann estimators were computed for each Mann–Whitney *U* test, along with corresponding 95% confidence intervals.

To test Exploratory Hypothesis 2, associations between trauma marker frequency and psychological symptom severity were examined using Spearman’s rank-order correlations (two-tailed). Correlations were computed between each of the three trauma marker indices (sum, density, coverage) and symptom severity scores derived from the HSCL-25 (anxiety, depression and total score) and the PCL-5 (total score). Analyses were conducted using available-case data (*N* = 27). For each symptom domain, the three trauma marker indices were treated as a single family of tests, and the Holm–Bonferroni correction was applied accordingly. Although directional hypotheses were specified, two-tailed tests were used to provide a conservative assessment of associations and to ensure consistency with two-sided bootstrap confidence intervals. Bias-corrected and accelerated (BCa) bootstrap confidence intervals (95%) were computed for correlation coefficients to provide robust estimates of uncertainty. Statistical significance was set at *p* < 0.05 after correction for multiple comparisons. Given the exploratory nature of the study and the hard-to-reach clinical population, no *a priori* power analysis was conducted; accordingly, results should be interpreted cautiously.

## Results

### Sample characteristics

For Hypothesis 1, the sample comprised 40 Afghan refugees who completed the attachment assessment. Participants were between 18 and 52 years of age (mean (mean) 25.43, s.d. = 7.20). The majority of the sample was male (75%, *n* = 30).

For Hypothesis 2, analyses were conducted in a subsample of 27 Afghan refugees with available symptom severity measures. Participants were between 18 and 52 years of age (mean 26.96, s.d. = 7.53). The majority of the sample was male (77.8%, *n* = 21). Most participants were unmarried (66.7%, *n* = 18). With regard to current occupation in Germany, 33.3% of participants were attending school (*n* = 9), 33.3% were unemployed (*n* = 9), 11.1% were employed (*n* = 3), 11.1% were attending language courses (*n* = 3) and 11.1% were engaged in childcare responsibilities (*n* = 3). Approximately one-quarter of the sample had entered Germany as unaccompanied minors (25.9%, *n* = 7).

Only a small proportion of participants held a residence permit (7.4%, *n* = 2), whereas the vast majority did not (92.6%, *n* = 25).

At treatment entry, medication data were available for 26 participants. Of these, the majority were receiving psychopharmacological medication (69.2%, *n* = 18), while 30.8% reported no psychopharmacological treatment (*n* = 8).

### Group differences in trauma marker expression by attachment classification

Of the 40 participants included in the analysis of Hypothesis 1, attachment representations were initially classified into the four standard AAP categories (secure, dismissing, preoccupied, unresolved). For the purpose of the present analyses, classifications were subsequently collapsed into unresolved (45%, *n* = 18) and resolved (55%, *n* = 22) groups. Descriptive statistics for the three trauma marker indices (sum, density and coverage) stratified by attachment group are presented in Table 1.

**Table 1:** Descriptive results of TM frequency by attachment group Table 1 long description.

TM Index	Mean (s.d.)		Median		Range	
	Resolved^a^	Unresolved^b^	Resolved	Unresolved	Resolved	Unresolved
Sum	4.18 (3.29)	16.28 (10.82)	4.00	16.50	0–11	3–39
Density	0.70 (0.75)	2.04 (2.26)	0.68	1.46	0–2.44	0–7.02
Coverage	2.05 (1.36)	4.56 (1.85)	2.00	5.00	0–5	1–7

*M*, mean; TM, trauma marker.

a. *n* = 22.

b. *n* = 18.

Across all indices, participants with an unresolved attachment representation showed substantially higher values, and all effects remained statistically significant after correction for multiple comparisons (Table 2).

**Table 2:** Mann–Whitney U test for TM frequency by resolved^a^ and unresolved^b^ attachment group Table 2 long description.

TM Index	*U*	*z*	*p* (two-tailed)	HLE (95% *CI*)	Effect size (*r*)
Sum	47.00	4.12	<0.001	11 [6, 15]	0.65
Density	42.00	4.24	<0.001	0.78 [0.45, 1.17]	0.67
Coverage	61.00	3.78	<0.001	3 [1, 4]	0.60

Effect sizes were interpreted as small (*r* = 0.10), medium (*r* = 0.30) and large (*r* = 0.50).

HLE, Hodges–Lehmann estimate; *r,* effect size estimate; TM, trauma marker; *U,* Mann–Whitney *U* statistic; *z,* standard score.

a. *n* = 22.

b. *n* = 18.

For the total number of trauma markers, the unresolved group displayed significantly higher values than the resolved group (mean ranks = 28.89 *v*. 13.64), Mann–Whitney U statistic (*U*) = 47.00, standard score (*z*) = 4.12, *p* < 0.001, effect size estimate (*r*) = 0.65. The Hodges–Lehmann estimator indicated a median difference of 11 TMs between groups (95% CI [6, 15]). Comparable patterns were observed for trauma marker density and coverage, with the unresolved group showing significantly higher values across both indices (all *p*s < 0.001, *r*s ≥ 0.60).

As expected, all participants classified as unresolved showed at least one trauma marker, because unresolved attachment classification in the AAP requires segregated systems material that remains unresolved.^8^ Importantly, unresolved attachment in the AAP is not defined by the presence of trauma markers, that is, segregated systems markers, per se. Rather, such material is evaluated according to whether it can be integrated or contained through organised attachment representations.^8^ Accordingly, attachment-related trauma markers – as applied in the present study – may occur across all attachment classifications. Consistent with this distinction, trauma markers were also present in the majority of participants classified as resolved, with 18 of 22 resolved individuals (81.8%) exhibiting at least one trauma marker.

Taken together, these findings provide strong support for Hypothesis 1, indicating that unresolved attachment representations are associated with a markedly higher frequency of attachment-related trauma markers in AAP narratives, both in absolute terms, relative to narrative length and in their distribution across attachment stimuli.

### Associations between trauma markers and psychological symptom severity

At treatment entry, participants showed high levels of psychological symptom severity. Mean scores on the HSCL-25 indicated elevated symptoms of anxiety (mean 3.04, s.d. = 0.51), depression (mean 3.09, s.d. = 0.60) and overall psychological distress (mean 3.07, s.d. = 0.52). All participants scored above the established HSCL cut-off of 1.75^45,46^ for anxiety, and 26 of 27 participants exceeded this cut-off for both depressive symptoms and overall psychological distress. PTSD symptom severity, as assessed by the PCL-5, was likewise high (mean 52.63, s.d. = 11.16), with all participants scoring above the clinical cut-off of 33.^47^ Symptom scores ranged from 1.70 to 3.90 for the HSCL-25 total scale and from 35 to 73 for the PCL-5.

To test Hypothesis 2 (Table 3), correlation analyses were performed separately for each of the three trauma marker indices (sum, density and coverage) and symptom severity measures derived from the HSCL-25 (depression, anxiety and total score) as well as the PCL-5 total score.

**Table 3:** Correlations between trauma marker frequency and symptom severity Table 3 long description.

Symptom severity	Spearman’s *r* [95% *CI*] for TM Index		
	Sum	Density	Coverage
HSCL^a^			
Anxiety	0.444 [0.157, 0.668]	0.403 [0.063, 0.668]	0.383 [0.033, 0.662]
Depression	0.507** [0.170, 0.726]	0.448* [0.100, 0.701]	0.486* [0.134, 0.751]
HSCL total	0.481* [0.128, 0.726]	0.400* [–0.021, 0.694]	0.460* [0.070, 0.763]
PCL-5^a^			
PTSD symptom severity	0.041 [–0.313, 0.409]	–0.031 [–0.404, 0.357]	0.103 [–0.291, 0.490]

All *p*-values are two-tailed; *p < 0.05; **p < 0.01 (after Holm–Bonferroni correction).

HSCL, Hopkins Symptom Checklist; PCL-5, Post-traumatic Stress Disorder Checklist for DSM-5; PTSD, post-traumatic stress disorder; *r,* effect size estimate; TM, trauma marker.

a. *N* = 27.

For anxiety symptoms as assessed by the HSCL-25, all three trauma marker frequency indices showed positive associations with symptom severity. Although these associations did not consistently remain statistically significant after correction for multiple comparisons, bootstrap confidence intervals suggested stable positive associations across indices.

Depressive symptom severity was consistently and robustly associated with trauma marker frequency across all three indices. These associations were moderate in magnitude and remained statistically significant after correction for multiple comparisons, indicating a reliable relationship between trauma marker expression and depressive symptoms.

Overall psychological distress, as measured by the HSCL-25 total score, was also positively associated with trauma marker frequency across indices. While most associations remained statistically significant after correction for multiple comparisons, one association (density) was characterised by greater uncertainty, as reflected in its confidence interval ( 0.021 to 0.694), and should therefore be interpreted cautiously.

In contrast, no significant associations were observed between trauma marker frequency and PTSD symptom severity as assessed by the PCL-5. Effect estimates were small and imprecise, and confidence intervals included zero, suggesting substantial uncertainty around these associations.

Taken together, these findings provide partial support for Hypothesis 2. Attachment-related trauma markers were robustly associated with depressive symptoms and general psychological distress, showed weaker and less consistent associations with anxiety symptoms and were not associated with PTSD-specific symptom severity.

## Discussion

### Summary of main findings

The present study investigated the narrative expression and the frequency of attachment-related traumatic content in a clinical sample of Afghan refugees receiving out-patient psychological treatment. Building on previous work focusing on categorical attachment classifications, this study adopted a dimensional, narrative-based approach by quantifying attachment-related trauma markers and examining their associations with attachment representations and psychological symptom severity.

Consistent with Hypothesis 1, participants classified as having an unresolved attachment representation exhibited a markedly higher frequency of attachment-related trauma markers across all indices compared to those with resolved attachment representations. This pattern was robust across absolute counts, marker density relative to narrative length and the distribution of trauma markers across attachment stimuli, indicating that unresolved attachment is associated with a more pervasive and intense narrative expression of attachment-related trauma.

Importantly, attachment-related trauma markers were not exclusive to unresolved attachment representations, but also occurred in the majority of participants classified as resolved. The pronounced group differences are therefore unlikely to reflect methodological circularity. Rather, they indicate that unresolved attachment is associated with a more pervasive and intensified narrative expression of attachment-related trauma, evident at a quantitative, content-based level beyond categorical classification.

With respect to Hypothesis 2, trauma marker expression was differentially associated with domains of psychological symptom severity. Higher frequencies of attachment-related trauma markers were robustly associated with depressive symptoms and overall psychological distress, whereas associations with anxiety symptoms were noticeably but non-significant. In contrast, no meaningful associations were observed between trauma marker expression and PTSD-specific symptom severity.

Taken together, these findings suggest that attachment-related trauma markers in narrative responses capture clinically relevant aspects of internalising distress and general symptom burden. At the same time, the absence of associations with PTSD symptom severity warrants further consideration.

### Results in the context of attachment theory

The finding that attachment-related traumatic content occurred more frequently at the narrative level in individuals with unresolved compared to resolved attachment representations is consistent with previous research in refugee populations. Prior studies have shown that traumatic flight experiences are reflected in AAI narratives in the form of unresolved discourse patterns.^48^ Another study similarly reported content-related similarities in AAI transcripts of refugees with unresolved attachment representations, including themes such as danger, abuse, loss and disturbing or frightening material.^49^

The present finding related to the first exploratory hypothesis provides initial evidence that attachment-related traumatic burden is not only observable at a categorical level but also at a quantitative, content-based level. It further suggests that the theoretical assumption that higher levels of traumatic burden are reflected in a greater frequency of traumatic content in narratives^28^ may also apply to refugee populations.

However, it should be noted that the only two previous studies examining differences in PTSD symptom severity between unresolved and resolved attachment representations did not find significant group differences, partly due to very high symptom severity in both attachment groups and resulting ceiling effects.^7,18^ Recent meta-analytic evidence confirms high prevalence rates of mental disorders in refugee and asylum-seeking populations.^50^ At the same time, refugees constitute a population that is particularly difficult to recruit,^51^ not least due to existing fears of stigmatisation in the Afghan community.^52^ Against this background, trauma marker frequency may serve as a sensitive indicator for differentiating attachment-related traumatic content at the narrative level.

The finding that trauma marker frequency was associated with depressive symptoms and overall psychological distress, but not with PTSD symptom severity, may appear paradoxical at first glance. Rather than indicating an anomalous finding, however, this pattern may suggest that trauma markers capture diffuse forms of attachment-related and trauma-related distress shaped by chronic adversity and ongoing post-migration stressors, instead of mapping narrowly onto PTSD-specific symptom severity. In the present sample, PTSD symptom severity was very high across participants, with all participants scoring above the clinical PCL-5 cut-off. This restricted range may have limited the ability to detect associations between trauma marker frequency and PTSD symptom severity.

Beyond this methodological explanation, the pattern may also reflect the clinical structure of distress in Afghan refugee populations. From an Afghan mental health perspective, interventions narrowly focused on symptom reduction – particularly PTSD – may overlook substantial sources of psychological burden. PTSD therefore represents an important, but not exclusive, outcome among interrelated forms of psychological suffering, including depression, anxiety, psychosocial impairment and culturally specific expressions of distress.^53^ Consistent with this, war-related traumatic stress in Afghanistan has been shown to be no more strongly associated with PTSD than with depression, anxiety or general psychological distress.^27^

The association between trauma markers and depressive symptoms should therefore not be interpreted as reflecting past traumatic experiences alone. In refugee populations, attachment-related themes such as abandonment, isolation, helplessness, failed protection and constriction may also be shaped by ongoing post-migration stressors and current social adversity. Refugees are exposed to multiple stressors before, during and after migration, including conflict, precarious transit conditions, family separation, poor living circumstances and barriers to care, which can have lasting negative effects on mental health.^33^ In the present sample, such cumulative and ongoing stressors may have contributed to a diffuse form of attachment-related distress that was more closely reflected in depressive symptoms and general psychological burden than in PTSD-specific symptom severity.

This interpretation is also consistent with Afghan idioms of distress. Psychological distress in Afghanistan is commonly described through culturally specific idioms such as *jigar khun* (deep, loss-related sadness), *asabi* (overwhelm and nervousness resulting from chronic life stressors) or *fishar* (inner emotional pressure or exhaustion), reflecting a holistic, everyday form of distress closely linked to social adversity. Consistent with this, neither narrative data nor questionnaire data reveal a clearly delineated trauma syndrome, as PTSD symptoms are not narratively dominant and do not form a distinct pattern.^54^

From an attachment perspective, these experiential dimensions of depression and general distress – affective overwhelm, exhaustion and relational strain – may be understood within the broader framework of defensive exclusion, including deactivation, cognitive disconnection and segregated systems.^37^ As markers of segregated systems in AAP narratives, trauma markers may specifically index failures to integrate or contain attachment-related fear, helplessness, abandonment and vulnerability.^8^ This may explain why trauma marker frequency was more closely associated with depressive symptoms and general psychological distress than with PTSD-specific symptom severity.

### Culture-specific communication of mental health and attachment

To better understand the observed patterns of narrative trauma marker expression, particularly their association with general distress rather than PTSD-specific symptoms, it is necessary to consider culture-specific modes of communicating psychological and attachment-related experiences.

Specific modes of communication among Afghans regarding attachment-related traumatic experiences underscore the importance of culture and its influence on how psychological and emotional content is communicated.

In Afghan contexts, collectivistic social orientations may be particularly relevant for many individuals, although not uniformly so. Such orientations can involve close adherence to social norms, moral codes and the perceived interests of family and community, often out of concern about shame or disgrace.^55^ One illustrative concept in this regard is *guzaara*, which describes an attitude of endurance and social accommodation that emphasises patience, restraint, adaptability and cooperation in order to cope with difficult living conditions while preserving important social relationships.^56^

At the level of communication, these patterns among Afghan patients can be conceptualised using Edward T. Hall’s notion of high-context communication. In contrast to lower-context communication cultures such as Germany and the United States, high-context communication places less information in the explicit, coded part of the message and relies more on physical context and internalised cues.^57^

In high-context communication, meaning is conveyed primarily through implicit contextual cues such as non-verbal behaviour, shared cultural conventions, tone and silence rather than through explicit verbal content. This style, more common in collectivistic cultures, favours indirect, restrained and relationally sensitive expression aimed at preserving harmony and avoiding loss of face, with listeners expected to infer meaning from context. In contrast, low-context communication prioritises explicit verbal content, directness and clarity.^58^ This framework is used here as a heuristic and should not be understood as implying a uniform communication style among Afghan patients.

In the present study, attachment-related trauma markers were operationalised to capture both explicit phrases and brief, emotionally condensed narrative elements, including single words and narrative constrictions. Accordingly, trauma-related distress was frequently expressed in condensed or implicit narrative forms rather than in elaborated trauma accounts.

Against this background, interview-based attachment assessments such as the AAI or the AAP appear particularly suitable, as they assess attachment indirectly through narrative responses and thereby allow for greater contextual embedding. The findings of the present study further support this assumption, as attachment-related trauma was frequently expressed in condensed, implicit narrative elements that would likely remain inaccessible to purely symptom-focused or questionnaire-based approaches. However, this advantage can only be realised when cultural competence in communication is adequately taken into account. Effective clinical communication is facilitated by clinicians’ cultural awareness and competence.

Conversely, when cultural differences between clinicians and patients remain unnoticed, misunderstandings may arise, for example through stereotypical assumptions or misreading culturally shaped expressions of distress. Accordingly, a crucial first step in cross-cultural clinical work is the explicit acknowledgement of cultural differences, followed by their careful and contextually informed interpretation.^59^

### Clinical implications

The assessment of narrative data on attachment and attachment-related traumatic experiences can contribute to a broader clinical understanding of psychological distress among refugees. The observed associations between trauma markers, depressive symptoms and general psychological distress suggest that attachment-related trauma in Afghan refugees may capture clinically relevant forms of suffering that extend beyond PTSD-focused symptom models. This is particularly important because refugee mental health may be shaped by cumulative adversity across pre-migration, migration and post-migration phases, including ongoing post-migration stressors, rather than by clearly bounded traumatic events alone.^33^

Narrative, attachment-based approaches may therefore be a valuable complement to standardised questionnaires, especially in one-to-one clinical contexts in which distress may be communicated implicitly, relationally or in culturally contextualised ways. Whereas standardised questionnaires rely on the explicit articulation of internal states, narrative methods allow for more contextualised, implicit and relational expressions of psychological distress. In such contexts, clinicians may facilitate treatment engagement by providing transparent information about available services, explaining links between somatic symptoms and psychological distress, actively addressing stigma, acknowledging potential inhibitions around emotional expression and considering patients’ preferred communication styles.^60^ This is particularly relevant when clinical encounters are interpreter-mediated, as the communication of implicit or emotionally condensed distress depends not only on the patient and clinician, but also on the linguistic and cultural mediation within the therapeutic setting.

The use of trained interpreters remains essential in refugee mental healthcare. Interpreters can support linguistic and cultural mediation and thereby facilitate clinical understanding and therapeutic engagement.^61^ At the same time, interpreter-mediated work requires a clearly established therapeutic frame, careful role clarification and adequate support for interpreters.^62^ These considerations are especially relevant for culturally sensitive attachment-focused assessment and treatment with Afghan refugees.

### Strengths and limitations

Several strengths of the present study should be highlighted. First, the study was conducted in a naturalistic out-patient treatment setting and included a clinically highly burdened and underrepresented population of Afghan refugees. This enhances the ecological validity of the findings and their relevance for clinical practice, particularly because the sample represents a relatively homogeneous refugee group, which is rarely the case in existing research. Second, the use of a narrative, attachment-based assessment enabled the examination of attachment-related trauma beyond categorical classifications and PTSD-focused symptom models. By introducing a dimensional coding of attachment-related trauma markers, the study offers a novel and clinically meaningful approach to capturing trauma-related distress as it is expressed in narrative form. This approach has the additional advantage of being sensitive to indirect, context-dependent information that may not be explicitly articulated in high-context communication cultures. Third, the study was conducted in a culturally sensitive manner, with assessments carried out in participants’ preferred languages and supported by trained interpreters where required.

Several limitations should also be considered. The sample size was modest, particularly for analyses involving self-report measures of symptom severity, which limits statistical power and the generalisability of the findings. Although the symptom subsample was nearly balanced with regard to attachment group and descriptive comparisons indicated no meaningful differences in age or gender between participants with complete and incomplete questionnaire data, the reduced availability of questionnaire-based symptom data remains an important limitation. Participants who were unable to complete questionnaires may have differed systematically from those included in the symptom analyses, particularly with regard to psychological distress, clinical stability, literacy or language-related barriers. Findings involving symptom severity should therefore be interpreted with caution.

A further limitation concerns trauma marker coding reliability. Although trauma marker identification was linked procedurally to the AAP coding evaluation underlying the final attachment classification, no separate interrater reliability coefficient was calculated specifically for trauma marker coding. Therefore, the reliability of the dimensional trauma marker indices cannot be evaluated independently of the reliability of the categorical AAP classifications.

Unfortunately, despite efforts to include both genders in equal proportions, Afghan women were less accessible than desired and comprised only 25% of the sample. This reflects a well-documented underrepresentation in research, which persists despite substantial evidence of high levels of psychological distress among Afghan women. It also highlights the need for greater attention to their health-seeking behaviours, which are often more complex and agentic than commonly assumed.^63^

Furthermore, the cross-sectional design captures attachment-related narratives and symptom severity at specific assessment points only and therefore precludes conclusions about temporal ordering or causality. The use of interpreters may have influenced the narrative data; however, interpreter-assisted assessments were conducted in accordance with established best-practice guidelines and reflect routine clinical practice in this context.

The interviewer’s positionality must also be considered when interpreting the findings. Although interpreter gender and cultural background were deliberately selected to balance potential power asymmetries, narrative expression – particularly among female participants – may still have been influenced by the interviewer’s gender and cultural background.

Finally, the present findings should be considered exploratory and require replication in larger and longitudinal samples. Future research should examine the temporal dynamics of attachment-related trauma markers and their sensitivity to therapeutic change, for example through pre–post treatment comparisons.

This exploratory study is among the first to examine how attachment-related traumatic experiences become narratively visible in Afghan refugees through the systematic identification of attachment-related trauma markers. The findings suggest that individuals with an unresolved attachment representation exhibit not only categorical differences but also quantitatively higher levels of attachment-related traumatic content in their narratives. In addition, trauma marker frequency was significantly associated with general psychological distress and depressive symptoms, whereas no associations were observed with anxiety or PTSD-specific symptom severity. Given the small sample size and exploratory cross-sectional design, these findings should be interpreted with caution. Nevertheless, they point to the potential added value of narrative, attachment-based assessment approaches, particularly in clinical encounters in which distress may be communicated implicitly, relationally or in culturally contextualised ways. Such approaches may enable trauma-related distress to be identified beyond narrowly defined PTSD symptom models. A culturally sensitive application of these methods, guided by cultural attunement and supported by well-trained interpreters, appears to be an important prerequisite for achieving an adequate clinical understanding in this context.

## Data Availability

The data that support the findings of this study are not publicly available due to their sensitive nature and the risk of participant identification, but may be made available from the corresponding author upon reasonable request and subject to ethical approval.

## Table 1 Long description

A table comparing trauma marker frequency by attachment group. The table has four rows and three columns. The columns are labeled Mean (s.d.), Median, and Range. The row labels are Sum, Density, and Coverage. Each row is further divided into Resolved and Unresolved groups. Row 1: Sum, Mean (s.d.): Resolved 4.18 (3.29), Unresolved 16.28 (10.82); Median: Resolved 4.00, Unresolved 16.50; Range: Resolved 0-11, Unresolved 3-39. Row 2: Density, Mean (s.d.): Resolved 0.70 (0.75), Unresolved 2.04 (2.26); Median: Resolved 0.68, Unresolved 1.46; Range: Resolved 0-2.44, Unresolved 0-7.02. Row 3: Coverage, Mean (s.d.): Resolved 2.05 (1.36), Unresolved 4.56 (1.85); Median: Resolved 2.00, Unresolved 5.00; Range: Resolved 0-5, Unresolved 1-7.

Navigate back to Table 1.

## Table 2 Long description

A table with four rows and five columns comparing trauma marker frequency across resolved and unresolved attachment groups using the Mann-Whitney U test. The columns are labeled TM Index, U, z, p (two-tailed), HLE (95% CI), and Effect size (r). The rows are labeled Sum, Density, and Coverage. Row 1: TM Index, Sum; U, 47.00; z, -4.12; p (two-tailed), <0.001; HLE (95% CI), 11 [6, 15]; Effect size (r), 0.65. Row 2: TM Index, Density; U, 42.00; z, -4.24; p (two-tailed), <0.001; HLE (95% CI), 0.78 [0.45, 1.17]; Effect size (r), 0.67. Row 3: TM Index, Coverage; U, 61.00; z, -3.78; p (two-tailed), <0.001; HLE (95% CI), 3 [1, 4]; Effect size (r), 0.60.

Navigate back to Table 2.

## Table 3 Long description

A table with five rows and four columns. The columns are labeled Symptom severity, Sum, Density, and Coverage. The rows are labeled HSCL, Anxiety, Depression, HSCL total, PCL-5, and PTSD symptom severity. The table presents Spearman’s r values with 95% confidence intervals for TM Index across different symptom severity measures. Row 1: HSCL, Sum: N/A, Density: N/A, Coverage: N/A. Row 2: Anxiety, Sum: 0.444 [0.157, 0.668], Density: 0.403 [0.063, 0.668], Coverage: 0.383 [0.033, 0.662]. Row 3: Depression, Sum: 0.507* [0.170, 0.726], Density: 0.448* [0.100, 0.701], Coverage: 0.486* [0.134, 0.751]. Row 4: HSCL total, Sum: 0.481* [0.128, 0.726], Density: 0.400* [-0.021, 0.694], Coverage: 0.460* [0.070, 0.763]. Row 5: PCL-5, Sum: 0.041 [-0.313, 0.409], Density: -0.031 [-0.404, 0.357], Coverage: 0.103 [-0.291, 0.490].

Navigate back to Table 3.
